# Developing Wound Dressings Using 2-deoxy-*D*-Ribose to Induce Angiogenesis as a Backdoor Route for Stimulating the Production of Vascular Endothelial Growth Factor

**DOI:** 10.3390/ijms222111437

**Published:** 2021-10-23

**Authors:** Serkan Dikici, Muhammad Yar, Anthony J. Bullock, Joanna Shepherd, Sabiniano Roman, Sheila MacNeil

**Affiliations:** 1 Department of Bioengineering, Izmir Institute of Technology, 35430 Izmir, Turkey; 2 Department of Materials Science & Engineering, Kroto Research Institute, University of Sheffield, Sheffield S3 7HQ, UK; a.j.bullock@sheffield.ac.uk (A.J.B.); s.roman@sheffield.ac.uk (S.R.); 3 Interdisciplinary Research Centre in Biomedical Materials (IRCBM), COMSATS University Islamabad, Lahore Campus, Lahore 54000, Pakistan; drmyar@cuilahore.edu.pk; 4 School of Clinical Dentistry, University of Sheffield, Sheffield S10 2TA, UK; j.shepherd@sheffield.ac.uk

**Keywords:** 2-deoxy-*D*-Ribose (2dDR), deoxy sugar, angiogenesis, wound healing, chronic wounds, wound dressing

## Abstract

2-deoxy-*D*-Ribose (2dDR) was first identified in 1930 in the structure of DNA and discovered as a degradation product of it later when the enzyme thymidine phosphorylase breaks down thymidine into thymine. In 2017, our research group explored the development of wound dressings based on the delivery of this sugar to induce angiogenesis in chronic wounds. In this review, we will survey the small volume of conflicting literature on this and related sugars, some of which are reported to be anti-angiogenic. We review the evidence of 2dDR having the ability to stimulate a range of pro-angiogenic activities in vitro and in a chick pro-angiogenic bioassay and to stimulate new blood vessel formation and wound healing in normal and diabetic rat models. The biological actions of 2dDR were found to be 80 to 100% as effective as VEGF in addition to upregulating the production of VEGF. We then demonstrated the uptake and delivery of the sugar from a range of experimental and commercial dressings. In conclusion, its pro-angiogenic properties combined with its improved stability on storage compared to VEGF, its low cost, and ease of incorporation into a range of established wound dressings make 2dDR an attractive alternative to VEGF for wound dressing development.

## 1. Background to Identifying 2dDR as a Pro-Angiogenic Sugar

2-deoxy-*D*-Ribose (2dDR) is a D-isomer of a deoxy pentose monosaccharide in which the hydroxyl group at the C-2 position is replaced by a hydrogen atom. 2dDR was discovered in 1930 by Phoebus Levene during his studies revealing DNA structure [1,2]. Since then, 2dDR is mostly known for its presence in DNA’s nucleic acid structure and naturally occurs in the body by the enzymatic degradation of thymidine to thymine via thymidine phosphorylase (TP) [3]. Phosphorylases catalyse the addition of a phosphate group from an inorganic phosphate to an acceptor. In the body, TP plays an important role in recovering nucleosides after DNA degradation. Although the reaction is reversible, TP’s function is primarily catabolic [4,5]. TP has an amino acid sequence identical to platelet-derived endothelial cell growth factor (PD-ECGF) [6,7] and, as previously stated, catalyses the phosphorylation of thymidine to thymine [8,9] and 2-deoxyribose-1-phosphate (2dDR1P). 2dDR1P will then be dephosphorylated within the cytoplasm to 2dDR, which is the form that can pass through the cell membrane. It has previously been shown that the addition of thymidine to platelets increases the levels of thymine and 2dDR but not 2dDR1P in the extracellular medium [4], which clearly shows that the phosphate is removed within the cytoplasm. Dephosphorylation allows the mobility of 2dDR from the cell membrane.

To understand the angiogenic potential of 2dDR, we need to learn about the pro-angiogenic potential of TP first. In the late 1970s, one of the remarkable discoveries about TP and angiogenesis was that elevated TP activity was found in cancer patients compared to controls [10]. Judah Folkman, a pioneer in the field of cancer angiogenesis, suggested that tumour growth is angiogenesis dependent [11], which explains why cancer patients showed increased TP and angiogenic activity.

In the early 1990s, TP was identified to have an identical sequence to PD-ECGF [6,7]. The angiogenic activity of TP is known to be dependent upon its enzymatic activity but research at the time could not explain its molecular mechanism. There were two popular opinions on why endothelial cells (ECs) were attracted (as an indicator of angiogenesis) to an area with TP enzyme activity; (i) the substrate (thymidine) for the enzymatic reaction or (ii) the side products were chemoattractant to ECs [12,13,14]. Since exogenous treatment of thymidine was not been found to have a positive effect on ECs, the latter option, catalytic production of 2dDR, as a side product of this enzyme activity, was considered to be the most promising reason for TP’s pro-angiogenic activity [5,15,16,17]. A few groups demonstrated a positive impact on proliferation and migration of ECs in response to 2dDR treatment [16,17].

Several groups then explored the effect of 2dDR at cellular and molecular levels in vitro. Essentially, 2dDR was shown to induce tubulogenesis [18,19], inhibit hypoxia-induced apoptosis [20], and increase VEGF and IL-8 production [21] of ECs in vitro supporting the early reports of 2dDR’s positive impact on proliferation and cell migration. All of these studies contributed significantly to describe the potential of 2dDR to be used in the world of pro-angiogenic drugs. However, none of these studies went beyond in vitro experiments and there was no attempt to establish any dose-dependent activity.

In 2017, our group became the first to investigate 2dDRs pro-angiogenic potential in a chick chorioallantoic membrane (CAM) bioassay, a useful in vivo assay that is approved by the U.S. Food and Drug Administration (FDA) for the pre-clinical evaluation of products for the management of chronic cutaneous ulcer and burn wounds [22] and widely used in angiogenesis studies [23,24,25,26,27,28], and also its effect on wound healing using a full-thickness skin wound in rats [29]. Dikici et al. demonstrated the dose-dependence of 2dDR and defined its active concentration range in the CAM bioassay [30] and on human aortic endothelial cells (HAECs) [31]. After determining an effective and safe dose range, several wound dressing products (the details of these products will be given in Section 5) were developed. These delivery vehicles were chitosan/collagen [29], Poly(3-hydroxybutyrate-co-3-hydroxyvalerate) (PHBV) [30], alginate [32,33], cotton fibres and cotton fibres that are coated with wax to reduce adhesion to wounds [34]. All of these studies clearly demonstrated that 2dDR is a pro-angiogenic deoxy sugar that can be easily introduced into a variety of biomaterials not only to stimulate angiogenesis but also to accelerate wound healing even in diabetic chronic wounds.

**Figure 1:**
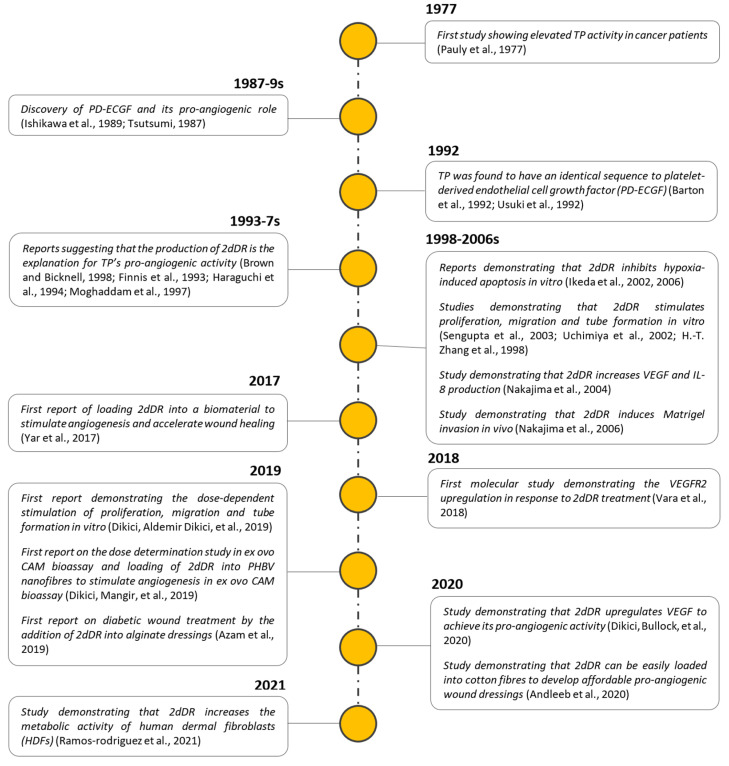
Key milestones of the exploration of 2dDR’s pro-angiogenic activity.

Figure 1 summarises the key milestones during the exploration of TP and 2dDR as potential pro-angiogenic agents.

To date, relatively few groups have explored 2dDR’s potential in the field of angiogenesis promoters as explained above. Thus, the literature on the angiogenic activity of 2dDR is not very comprehensive. Accordingly, the Web of Science (WoS) and PubMed databases were screened for articles in which 2dDR, TP and VEGF were studied for their angiogenic activity. Data were generated using “pro-angiogenic agent name (i.e., 2-deoxy-D-Ribose)” and “angiogenesis” as search terms. In the WoS database, only 27 studies were found studying the potential angiogenic effect of 2dDR to date, whereas there were 696 and 43603 studies on the angiogenic activity of TP and VEGF, respectively. Similarly, in PubMed database, there were 25 publications in which 2dDR was considered as a pro-angiogenic agent, whereas 463 and 43,517 studies were conducted on the pro-angiogenic activity of TP and VEGF, respectively.

**Figure 2:**
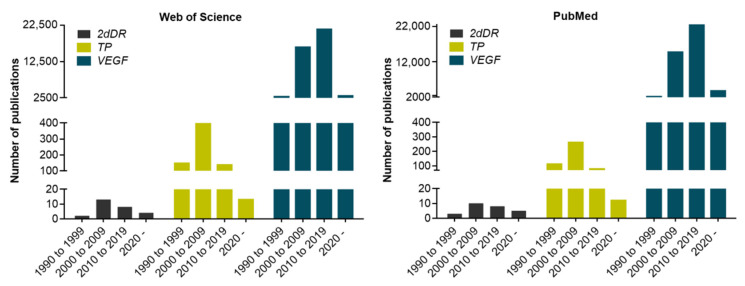
Distribution of angiogenesis-related publications using the WoS and PubMed databases on 2dDR, TP and VEGF from 1990 to date.

The distribution of these publications over the years in the WoS and PubMed databases from 1990 to the current time is given in Figure 2.

## 2. Exploration of the Dose-Dependent Biological Activity of 2dDR

Although a few groups have previously shown biological activity of 2dDR using in vitro models and in vivo models, a single dose or a maximum of two doses were selected in these studies. In 2019, Dikici et al. reported a dose-dependent response to 2dDR treatment.

In 1994, Haraguchi et al. reported that both 10 and 100 µM of 2dDR act as a chemoattractant to bovine aortic endothelial cells (BAE) on a fibronectin matrix, and a 50 pmol dose of 2dDR stimulates angiogenesis in the CAM bioassay [35].

In 1998, Zhang et al. reported that 2dDR, but not the enantiomer 2-deoxy-l-ribose (2dLR), stimulates the migratory response of microvascular endothelial cells (HMEC-1) using a Boyden chamber assay [36]

In 2002, Uchimiya et al. showed that both 10 and 100 µM of 2dDR stimulate migration of BAE in a modified Chemotaxicell chamber assay, and 10 µM of 2dDR enhances tube formation of BAE seeded on a type I collagen gel [18].

In 2003, Sengupta et al. demonstrated that 2dDR induces tube formation of human umbilical vein endothelial cells (HUVECs) at 1 µM concentration, using an AngioKit™ coculture system, and both 0.1 and 10 µM doses of 2dDR have a positive impact on the proliferation of HUVECs in a stretch wound healing assay. In addition to these in vitro results, they also showed increased an angiogenic activity in response to 2 nmol of 2dDR treatment in a sponge granuloma model [17].

In 2004, Nakajima et al. reported that 100 µM of 2dDR increases VEGF and interleukin-8 (IL-8) mRNA expressions in a human epidermoid carcinoma cell line (KB) under hypoxic conditions [21].

In 2006, Nakajima et al. demonstrated that 100 µM 2dDR increases the secretion and activity of matrix metalloprotease-9 (MMP-9) of human epidermoid carcinoma cells (KB cells) and upregulates the invasive behaviour of KB cells in a Matrigel invasion assay, where Matrigel serves as a reconstituted basement membrane for cells to degrade and invade [37].

In 2017, Yar et al. showed that 1 mg/mL 2dDR loaded into chitosan (CS)/collagen hydrogel promotes angiogenesis in the CAM bioassay and accelerates healing of cutaneous wounds in rats [29].

In 2018, Vara et al. reported that a 8 µM to 1 mM range of 2dDR upregulates VEGF-receptor 2 (VEGFR2) in HUVECs by activating NADPH Oxidase 2 (NOX2) and triggers Nuclear Factor Kappa B-dependent angiogenesis [38].

In 2019, Dikici et al. conducted a dose-dependent study of 2dDR in vitro. In this study, a range of 5 concentrations from 10 µM to 1 mM of 2dDR increased the metabolic activity and proliferation (assessed using resazurin reduction assay) and migratory response (assessed using a modified Boyden chamber assay) of HAECs. 100 µM of 2dDR was also found to stimulate tube formation (assessed using a Matrigel tube formation assay) of HAECs while 1 µM was ineffective and 10 mM showed toxic effects on these cells [31,39].

In 2019, Dikici et al. investigated the direct administration of 3 different doses of 2dDR into an ex ovo CAM bioassay. In this study, 20 and 200 μg/day/embryo applications of 2dDR induced angiogenesis, while 1000 μg/day/embryo dose was ineffective. In addition, 1 g PHBV dressings loaded with 250 and 500 mg of 2dDR showed significantly increased angiogenesis in the area of implantation of the dressing on the CAM [30].

In 2019, Azam et al. assessed the healing of skin wounds in vivo with commercially available alginate dressings containing 5% and 10% (*w*/*v*) of 2dDR using a diabetic rat wound healing model [33]. Both concentrations of 2dDR stimulated wound healing to a greater extent than the alginate dressing on its own.

In 2020, Andleeb et al. reported that loading of 5% (*w*/*v*) of 2dDR into cotton fibre dressings and into cotton-wax dressings stimulated angiogenesis in a CAM bioassay [34]. Cotton fibre dressings, a very commonly used dressing used worldwide, were used on their own to absorb exudate and coated with wax to reduce adhesion to wounds, such as in burns injuries.

In 2020, Dikici et al. showed that direct addition of 10 µM and 1 mM doses of 2dDR and the release of 2dDR from alginate dressings (10% (*w*/*v*) 2dDR per alginate dressing) into the growth media increased VEGF production from HAECs (evaluated using Human VEGF ELISA MAXTM Deluxe Set) [32].

In 2021, Ramos-Rodriguez et al. demonstrated that 50 to 1000 µM of 2dDR added directly to the culture media increased the metabolic activity of human dermal fibroblasts (HDFs) over 6 days (assessed by resazurin reduction assay) [40].

To sum up, from the 1990s to now, relatively few groups have explored 2dDR’s pro-angiogenic potential. Thus, the literature is not very comprehensive and until 2019, there were no studies conducted to establish a dose-dependent response to 2dDR. Research from the MacNeil/Yar groups demonstrated the effective doses of 2dDR using established in vitro and in vivo assays. However, the mechanism of action of 2dDR has not been clarified completely. Current data suggest that 2dDR triggers upregulation of VEGF production but further studies are needed to get a full picture in this area as will be discussed shortly.

## 3. What Do We Understand of the Mechanism of Action of 2dDR? How Does Sugar Structure Affect Function?

In peptide biology, it is well understood that not only molecular composition but 3D structural architecture to a large extent dictate function usually by interaction with receptors of precise 3D conformation. Unlike proteins, carbohydrates can take on several conformations depending on surrounding solvents and temperature. Before reviewing the published often contradictory literature on the pro-and anti-angiogenic effects of several sugars close to 2dDR we present a summary of the current understanding of the conformations of how 2dDR and related small sugars can be found.

### Isomers and Conformational Structures of Deoxyribose

**Figure 3:**
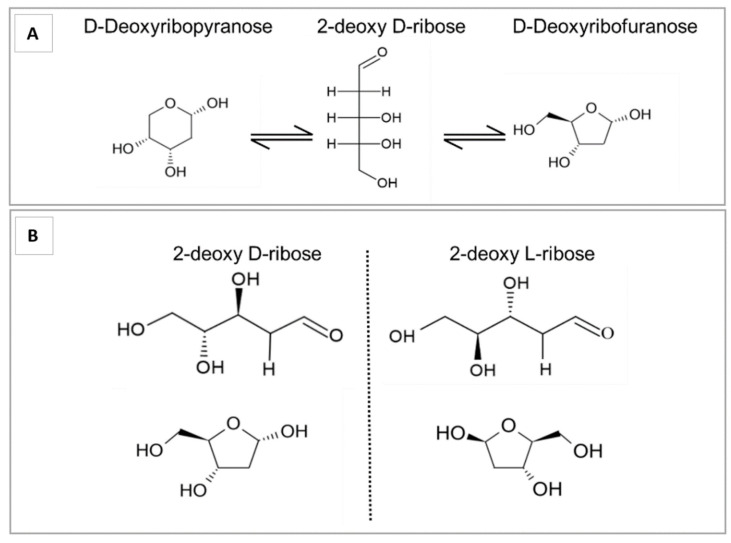
(**A**) Tautomeric forms of 2dDR in aqueous solution. 2dDR can exist as deoxyribofuranose, deoxyribopyranose and an intermediate chain form. (**B**) Stereoisomers of 2-deoxyribose. The asymmetric chiral carbon at position 2 leads to the existence of D and L isomers.

In an aqueous solution, deoxyribose exists as a tautomer of 3 anomers—a 5 membered ring deoxyribofuranose, a 6 membered ring deoxyribopyranose form and an intermediate chain form—described by Lemieux [41]. The relative amounts of each form are dependent on the solvent and temperature. In an aqueous solution at room temperature, this is 75% deoxyribofuranose, 25% deoxyribopyranose, and 0.7% chain form (Figure 3A).

Deoxyribose is also an enantiomer, existing in two stereoisomers, the biologically active d-form, and the rarer l-form. Like the d-form, the l-form exists in solution as furanose, pyranose, and chain forms (Figure 3B). While they have the same chemical formula and the same physical properties, their interactions with other chemical groups, proteins and enzymes differ.

The D-form is a structural component of DNA, the lack of a 2′ hydroxyl group lending the structure conformational flexibility (compared to ribose in RNA) and allowing the formation of the double helix. 2dDR has been shown to be biologically active in the promotion of VEGF-mediated angiogenesis although it is not clear which form of the D-anomers of 2dDR is the biologically active form responsible for the angiogenic activity.

The biological inactivity of the L-form lends itself to the study of inhibition of 2dDR-mediated functions, for example, the L-form inhibits the protection afforded by 2dDR against hypoxic-mediated apoptosis via interaction with thymidine phosphorylase [37]. While the D-form of 2dDR is a precursor for DNA synthesis, the L-form is used to create L-DNA (mirror DNA), which can store information identical to normal DNA but is resistant to biodegradation [42].

## 4. Pro- and Anti-Angiogenic Activity of Small Sugars

In contrast to the literature on the pro-angiogenic effects of 2dDR, the reports on other small sugars are very mixed as detailed below.

***D-glucose (DG):*** There are conflicting reports demonstrating the pro- and anti-angiogenic properties of DG in the literature. In 1993, Vogel et al. studied the migration of bovine corneal endothelial cells (BCE) in response to several small sugars using a modified Boyden chamber assay and reported migration toward DG (44–88 mM) [43]. Similarly, in 1999, Shigematsu et al. reported that 7–23.7 mM doses of DG stimulated migration and tube formation of HUVECs [44]. In 2016, Madonna et al. suggested that high glucose (30.5–50.5 mM) induces cyclooxygenase (COX-2) expression and the migration (assessed by CytoSelect™ cell migration assay) and tube formation (assessed by Matrigel tube formation assay) capability of HAECs [45]. In contrast to these positive reports, in 1999, Teixeira et al. demonstrated that daily injections of 22 mM of DG inhibited angiogenesis in the rat sponge model [46]. Similarly, in 2012, Jiraritthamrong et al. showed that 10.4–16.2 mM concentrations of DG inhibited the vessel-forming capacity of cultured endothelial progenitor cells (EPCs) using a Matrigel tube formation assay [47].

***2-deoxy-L-Ribose (2dLR):*** The literature on 2dLR is also contradictory. In 2002, Uchimiya et al. reported that 100 µM 2dLR could reduce the migratory response (assessed using Chemotaxicell chambers) and tubulogenesis (on type I collagen gel) of BAE, and 200 ng of 2dLR inhibits TP-induced angiogenesis in a rat corneal assay [18]. Ikeda et al. showed that 10–50 µM doses of 2dLR promoted hypoxia-induced apoptosis of HL-60 cells [48]. Nakajima et al. reported that 10–100 µM of 2dLR inhibits both mRNA levels and secretion of IL-8 and VEGF by KB cells [21] and 100 µM 2dLR inhibits Matrigel invasion of tumours by suppressing MMP-9 in nude mice [37]. Although all these studies are consistent on the anti-angiogenic activity of 2dLR, in 2003, Sengupta et al. also suggested that 2 nmol of 2dLR promotes an angiogenic response in a sponge granuloma model of angiogenesis [17]. Similarly, Yar et al. demonstrated the promotion of angiogenesis by the release of 2dLR (1 mg/mL) from chitosan/collagen hydrogels in a CAM bioassay [29].

***2-deoxy-D-Glucose (2dDG):*** Reports on 2dDG are quite consistent in its anti-angiogenic activity. In 2008, Tagg et al. showed that 1–100 mM of 2dDG inhibited the proliferation of MCF-7 (breast) and LNCaP (prostate) cancer cell lines, and 3 mM 2dDG reduced ATP levels [49]. In 2010, Merchan et al. reported the inhibition of proliferation, migration and tube formation capability of HUVECs by 0.06 to 6 mM doses of 2dDG which also inhibited angiogenesis in vivo when administered a concentration of 6 mM (assessed by Matrigel plug assay) [50]. In 2015, Chuang et al. demonstrated that 0.05 to 1 mM doses of 2dDG inhibited the tube formation (on ECMatrix^TM^ coated well plates) of both HUVECs in vitro and in rat aortic rings. 2dDG (0.1 to 1 mM) also reduced cell invasion and migration in HUVECs as well as the mRNA and protein expression of MMP-2 [51]. In 2016, Kovacs et al. reported the downregulation of AKT and ERK pathways and inhibition of tube formation (assessed by Matrigel tube formation assay) of Human microvascular endothelial cells from lung (HMVEC-L) in response to 0.6 mM 2dDG [52]. In 2016, Zhang et al. demonstrated that 2dDG has a negative impact on proliferation (2.5–40 mM doses—assessed by Cell Counting Kit-8 assay) and migration (5 mM dose—assessed by Transwell Permeable Supports) of two colorectal cancer cell lines (HCT116, LoVo) [53]. In 2019, Singh et al. showed that 0.625 to 25 mM doses of 2dDG impaired the metabolic viability (assessed by MTT), migration (assessed by scratch assay) and tube formation (assessed by Matrigel tube formation assay) ability of HUVECs, and Matrigel plugs loaded with 0.2% and 0.4% (*w*/*v*) of 2dDG significantly reduced neovascularisation when implanted into the C57BL/6 mice [54].

In summary, DG is reported to have both pro-and anti-angiogenic activity, 2dDG is consistently reported to be anti-angiogenic and 2dLR has reports of both pro-and anti-angiogenic activity. As 2dDR is predominantly pro-angiogenic, we suggest that it is challenging to draw any simple relationship between sugar structure and angiogenic activity.

## 5. Developing Dressings Containing 2dDR to Stimulate Angiogenesis

In addition to the studies to demonstrate the pro-angiogenic properties of 2dDR, in recent years, a considerable progress has been made in developing dressings to stimulate angiogenesis by loading 2dDR into a range of carriers.

There have been decades of work developing dressings to stimulate angiogenesis to accelerate wound healing, particularly in those wounds which are slow to heal. Predominantly such wounds are found in the elderly or in those with diabetes, which compromises the development of small blood vessels which play a major role in wound healing [27].

As expected, there are a wealth of studies seeking to deliver well-known pro-angiogenic stimulators such as VEGF or PDGF or more recently, in work from our own and many other laboratories, delivering heparin which can act to bind local pro-angiogenic growth factors [55,56].

Increasingly questions have moved from “Do these work “to “Do these work sufficiently at a production cost, which makes them clinically attractive”.

In treating chronic wounds which may persist for many months key questions which need to be asked are “is this safe?”, “does it have any adverse effects on healing?”, “does it deliver significant clinical benefit to the rate of wound healing?”, “is it convenient to use for the patient and the wound care nurse?”, “is it economically attractive compared to other established wound care dressings?”.

As an example, there are many studies that show that local delivery of VEGF can stimulate wound healing, but this peptide is relatively unstable and dressings need to be applied at frequent intervals. Delivery of this peptide is expensive and dressings must be stored appropriately before use. Many published studies show a potential clinical benefit but have not been translated into dressings taken up for routine use.

One exception is the hydrogel product containing PDGF-Becaplermin, which is used in some diabetic foot ulcer clinics. It has a modest stimulatory effect on wound healing but can be easily stored in the fridge.

Pharmaceutical companies vigorously pursued the development of dressings delivering VEGF but these have not become routinely adopted in the clinic.

Mindful of this background, we were attracted to explore the development of dressings releasing 2dDR, as they offered the potential for an effective, safe and conveniently used dressing for wound care. A conventional wound dressing should create a temporary protective barrier, absorb wound exudate, and preserve a moist environment to support re-epithelialisation [33,34,57]. Conventional, inexpensive, and highly absorbent dressings for treating simple wounds do not participate actively in the healing process [57]. However, the management of deep and chronic wounds that fail to progress through the physiological healing process in a timely manner requires bioactive dressings that promote wound healing [58,59].

Accordingly, our group have explored the addition of 2dDR to simple dressing materials as an affordable and effective way of developing bioactive dressings. The dressings our group have explored to date are summarised below.

**Figure 4:**
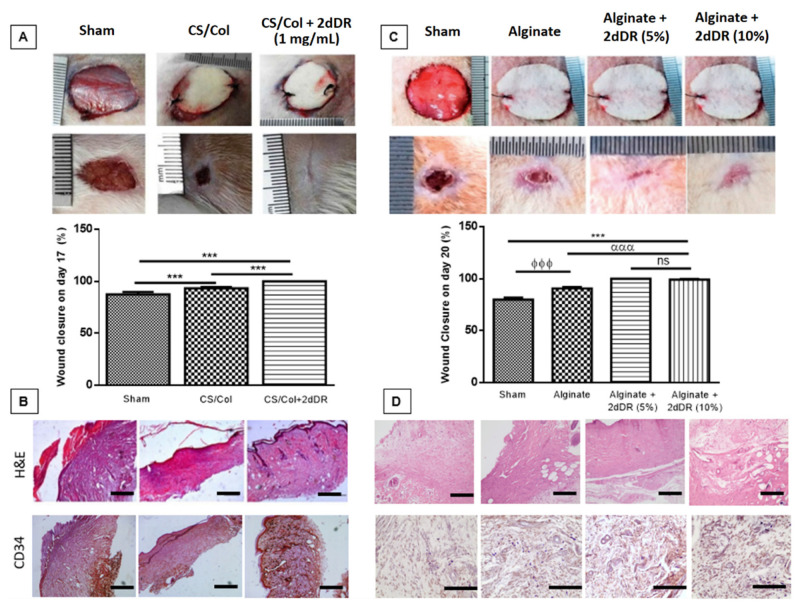
Gross, histological and graphical representation of wound closure analysis of animal studies. (**A**) Macroscopic analysis and (**B**) H&E staining and immunostaining with CD34 marker of the wounds showing the healing of cutaneous wounds in 17 days when treated with 2dDR-loaded CS/Collagen hydrogels (n = 3). (**C**) Macroscopic analysis and (**D**) H&E staining and immunostaining with CD34 marker showing the healing of diabetic wounds in 20 days when treated with 2dDR-loaded alginate dressings (n = 4). Scale bars represent 100 µm. Results are presented as mean ± SD where *** *p* < 0.001, ^ϕϕϕ^ *p* < 0.001, ^ααα^ *p* < 0.001, ^ns^ *p* > 0.05 (not significant). Images, used in (**A**,**B**), are adapted from [29] with permission granted by Materials Today Communications, Copyright 2017 Elsevier. Images, used in (**C**,**D**), are adapted from [33] with permission granted by Journal of Biomaterials Applications, Copyright 2019 Sage Publications.

***Chitosan/collagen hydrogels:*** Yar et al. developed 2dDR-releasing CS/collagen hydrogels that were crosslinked by using 4% triethyl orthoformate and loaded with 1 mg/mL of 2dDR. They confirmed the success of 2dDR loading via Fourier Transform Infrared Spectroscopy (FTIR). 2dDR-releasing CS/chitosan gels promoted angiogenesis in the CAM bioassay within 7 days, and accelerated the healing of wounds in a rat model in 17 days, whereas untreated and control gel-implanted wounds remained unhealed (Figure 4A,B). Immunohistochemistry analysis of wounds treated with 2dDR showed a good level of neovascularisation assessed by staining for ECs using a CD34 antibody. In addition, CD80 (against M1 macrophages) and CD163 (against M2 macrophages) immunostaining showed a slight dominance of M2 macrophages activity compared to M1 on day 17, as an indication of constructive remodelling and a lower inflammatory response, when the wounds were treated with 2dDR-loaded dressings [29].

***PHBV fibres:*** In 2019, Dikici et al. loaded 250 and 500 mg of 2dDR into 1 g of PHBV dressings and showed its release over 30 days using ultraviolet (UV) spectrophotometry at 238 nm wavelength. Approximately 90% of the 2dDR was found to be released from PHBV fibres within 3 days and stimulated angiogenesis in the ex ovo CAM bioassay [30].

***Alginate dressings:*** Azam et al. incorporated 5% or 10% (*w*/*v*) of 2dDR into commercially available alginate dressings and showed that almost all 2dDR was released in 3 days. They reported that these 2dDR-releasing alginate dressings facilitated healing of chronic wounds in 20 days using a diabetic rat wound healing model [33] (Figure 4C,D). Both concentrations of sugar were equally effective in support of these findings, in 2020, Dikici et al. showed that 2dDR-releasing alginate dressings stimulated VEGF production of HAECs in 7 days [32].

**Cotton-wax dressings:** Andleeb et al. used the 5% loading concentration of 2dDR to develop accessible and affordable pro-angiogenic dressings. In this study, two different carrier dressings were tested; a non-woven cotton dressing which is highly absorbent, designed to absorb excessive exudate absorption, and relatively less sticky to wounds; and a wax coated cotton dressing which is frequently used as first aid treatment for burn wounds. They reported several methodologies to optimise the release of 2dDR at an effective concentration range from both of these non-woven and wax-coated cotton dressings, and demonstrated that 5% 2dDR-loaded cotton-wax and non-woven dressings induced neovascularisation in the CAM bioassay [34].

## 6. Understanding the Mechanism of Action of 2dDR

In contrast to VEGF, to date, only a limited number of groups have studied the mechanism behind the pro-angiogenic activity of 2dDR.

In the 2000s, researchers proposed a potential mechanism that is based on the induction of oxidative stress due to the endogenous generation of 2dDR by TP activity which catalyses the degradation of thymidine to thymine. This has been considered as a potential way to stimulate the production and secretion of several pro-angiogenic signal proteins and chemokines such as VEGF and IL-8. They were thought to be internalised by the ECs to trigger the pro-angiogenic cascade [17,19,60].

In 2018, Vara et al. approached this question from a different angle and investigated the upregulation of VEGFR2 in response to 2dDR treatment. They suggested that (i) the 2dDR-1-phosphate (2dDRP) could be produced endogenously by cells that have been shown to have TP activity (i.e., macrophages, platelets and cancer cells) and then secreted to the extracellular environment or (ii) TP might be released from cells due to the disruption of the cell membrane (injuries) to act extracellularly on the degradation of thymidine to thymine and generation of 2dDR extracellularly. After one of the two suggested production mechanisms, 2dDR can be taken up by ECs and activates NOX2 that will act on NF-κB to upregulate VEGFR2. Their results demonstrated that 2dDR drives VEGF-dependent angiogenesis via upregulation of VEGFR2 on the cell membrane [38].

In 2020, our group set up an experiment to evaluate whether 2dDR could potentially increase the production of VEGF by the ECs. In this study, HEACs were treated with several doses of 2dDR, and VEGF in the growth media was quantified. Our results showed that 2dDR was the only sugar that increased VEGF production. Other small sugars including DG and 2dLR did not promote the production of VEGF by HAECs [32].

To the best of our knowledge, 2dDR achieves its pro-angiogenic activity via VEGF-dependent angiogenesis. This knowledge is particularly important with respect to clinical translation of the 2dDR-releasing pro-angiogenic wound dressings, since exogenous use of VEGF in an uncontrolled manner raises safety concerns for the production of large leaky blood vessels such as are observed in tumour angiogenesis [60,61,62,63]. Regarding the structure–activity relationship, our knowledge on why 2dDR is the only sugar that stimulates VEGF production of ECs but no other sugars is very limited and it needs further clarification. In such cases, an approach of in silico computer-aided drug design (CADD) might be used to identify potential active drug molecules. Such in silico (computational) studies may be helpful to understand the mode of interaction of 2dDR with target proteins and to propose some other 2dDR analogues that could be potential candidates with the potential to stimulate (or inhibit) angiogenesis. This would require a team of synthetic organic chemists, biologists and computational scientists and is beyond our expertise.

## 7. The Stability of 2dDR

One of the disadvantages of peptide growth factors is that they become very unstable when prepared as solutions. VEGF has been reported to lose approximately 60% and 70% of its activity over 12 and 30 days, respectively [64]. In the manufacturer’s product information sheet (Sigma Aldrich, Cat. No. V7259), it is stated that “reconstituted VEGF can be stored at 2–8 °C for up to one week”.

To assess the stability of 2dDR, only two stability tests have been performed to date in the presence and absence of cells.

In the first study, the stability of the aqueous solution of 2dDR at room temperature was evaluated by Azam et al. The results demonstrated that 2dDR was stable with no significant change recorded in the amount of 2dDR over two weeks [33]. The second experiment was conducted to study the stability of 2dDR in the presence or absence of HAECs. The results demonstrated that there was a significant decrease in the amount of 2dDR present in the growth medium over 14 days (both in cellular and acellular conditions) at 37 °C. The decrease in the amount of 2dDR was higher when HAECs were present. This can be explained by the internalisation of 2dDR by cells to be used to increase VEGF production. By day 14, approximately 40% and 30% of the total 2dDR had disappeared from the growth medium, respectively in the presence and absence of HAECs [32].

2dDR stability in alginate dressings was also evaluated after gamma sterilisation (irradiation dosage of 25 KGy) by Azam et al. It was noted that 2dDR did not undergo any chemical structural change/decomposition or reaction with alginate dressings due to gamma treatment. Almost all of the 2dDR loaded (>90%) was released either from gamma sterilised or non-sterilised alginate dressings [33]. Similarly, Andleeb et al. later reported that 2dDR was stable and gamma sterilisation did not affect its release from cotton dressings [34].

The stability studies of 2dDR are summarised in Table 1.

## 8. The Pro-Angiogenic Potential of 2dDR Compared to VEGF

VEGF is an established pro-angiogenic factor that has a prime role in inducing angiogenesis [65]. To date, the mechanism of action of VEGF has been studied widely, and we have previously summarised its main pathways that regulate angiogenesis [32]. ECs have been shown to be sensitive to VEGF, and the binding of VEGF to its receptor triggers the cascade that regulates proliferation, migration and survival of ECs [66,67].

VEGF is the gold standard for the induction of vascularisation. However, there are major concerns raised over its safety. The use of exogenous VEGF without controlled delivery systems has been shown to lead to the formation of extremely leaky [61], permeable [62] and haemorrhagic [60] vessels which are associated with tumour development. Please note there is no evidence that VEGF induces tumours but such blood vessels could assist growth and metastasis of existing tumours. Our group alongside many others have developed biomaterials to bind heparin in an attempt to bind and release VEGF in a controlled manner [55,56]. However, introducing VEGF to biomaterials requires the use of multistep coating techniques, experienced users and is time-consuming and expensive. Instead of the use of exogenous VEGF, the upregulation of endogenous VEGF production via administration of 2dDR merits exploration.

In addition to raised concerns over safety, use of exogenous VEGF is a very expensive approach similar to the use of other recombinant growth factors. For instance, when the pro-angiogenic doses were taken as 80 ng/mL and 134 µg/mL (1 mM), respectively for VEGF (catalogue number: V7259) and 2dDR (catalogue number: 121,649), then 1 L of 2dDR solution at 1 mM is approximately 2000 times less expensive than 1 L of VEGF solution at 80 ng/mL. These prices were taken from the Sigma Aldrich catalogue.

Accordingly, we asked to what extent did this sugar mimic the biological effects of VEGF.

**Figure 5:**
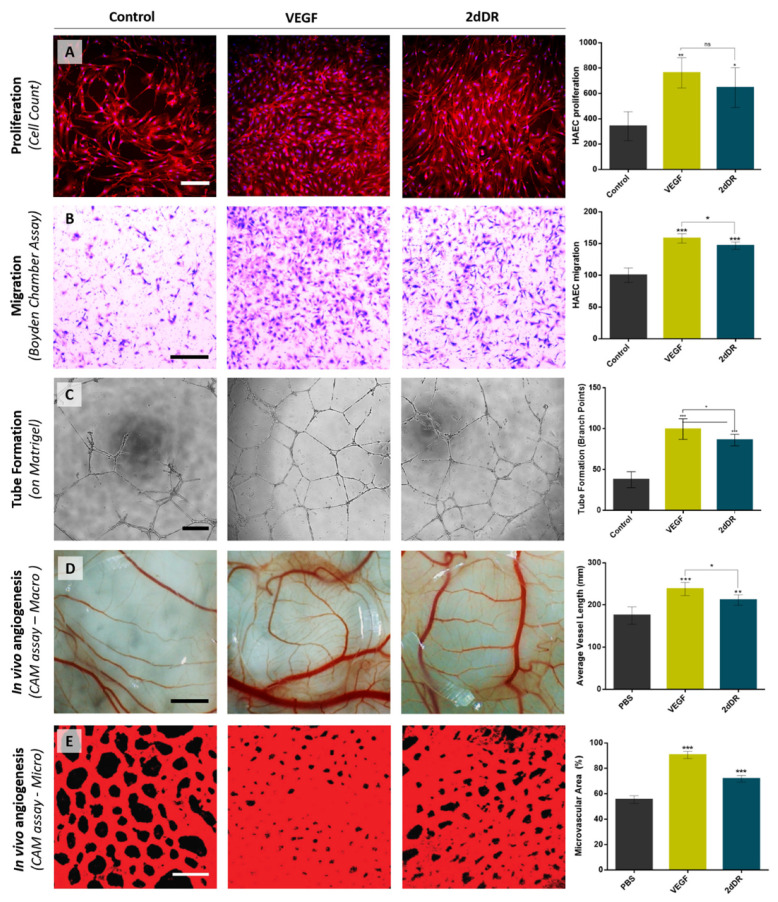
Representative images demonstrating the pro-angiogenic potential of 2dDR compared to VEGF in terms of stimulating the (**A**) proliferation, (**B**) migration, (**C**) tube formation of HAECs, and in vivo angiogenesis assessed by (**D**) macro vessels and (**E**) microvasculature in ex ovo CAM bioassay. (*** *p* ≤ 0.001, ** *p* ≤ 0.01, * *p* ≤ 0.05, ns: not significant, n = 3). Scale bars represent 200 μm for proliferation, 250 μm for migration and tube formation images, 2 mm for macro and 50 μm for confocal microvessel images of CAM bioassay. Images, used in (**A**,**D**), are adapted from [32] with permission granted by Microvascular Research, Copyright 2020 Elsevier. Images, used in (**E**), is adapted from [30] with permission granted by Regenerative Medicine, Copyright 2019 Future Medicine.

In 2019, Dikici at al. reported the effects of 2dDR on the proliferation (Figure 5A), migration (Figure 5B) and tube formation (Figure 5C) of HAECs compared to VEGF. The results of this study showed the potential of 2dDR in increasing the proliferation, migration and branching capability of HAECs being approximately 95%, 94%, 87% as effective as VEGF, respectively [31].

In 2019, a 2dDR dose study using an in vivo CAM assay was performed with VEGF as positive control. The results demonstrated that treatment with 2dDR was almost 80% as effective as VEGF in the stimulation of new blood vessel formation [30].

In 2020, the change in the metabolic activity of HAECs in response to 2dDR and VEGF treatment was investigated, and 2dDR was found to be approximately 90% as potent as VEGF in increasing the metabolic activity of HAECs. In the same study, the daily application of 2dDR solution to CAM augmented the total vessel length, which was roughly 90% of the effect achieved by VEGF administration (Figure 5D). Similarly, treatment with 2dDR was found to be approximately 80% as effective as VEGF in increasing the percentage area covered by microvasculature in the drug administration zones on CAM (Figure 5E) [32].

## 9. Ongoing Work

### 9.1. 2dDR and Skin Microbiology

**Figure 6:**
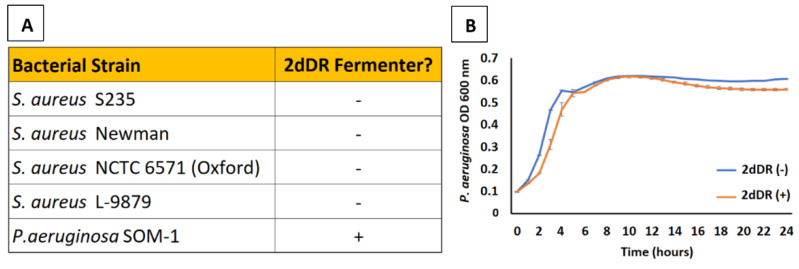
(**A**) Among the four strains of *S. aureus* tested, none were capable of fermenting 2dDR whilst the *P. aeruginosa* strain was a fermenter of 2dDR [29]. (**B**) Growth curves measuring optical density (absorbance at 600 nm) of *P. aeruginosa* SOM-1 over 24 h in media supplemented with and without 2dDR reveal that there is no effect of growth of this strain. Error bars represent the SEM (n = 3).

When preparing a topical dressing for skin wounds, it is advisable to use components that will not perturb the surrounding skin microbiota or cause a dysbiosis of the microbes either surrounding or within the wound, leading to overgrowth of pathogenic species. Given that 2dDR is a potential carbohydrate source for bacteria, Yar et al. previously published a pilot study investigating whether two species of bacteria commonly found in chronic skin wounds, * Pseudomonas aeruginosa* and *Staphylococcus aureus*, were able to metabolise 2dDR. Of the four strains of *S. aureus* (S235, NCTC 6571 (Oxford), Newman, and L-9879) and one of *P. aeruginosa,* none of the *S. aureus* strains appeared to metabolise the 2dDR (Figure 6A) [29]. Similarly, it has been previously reported that only a very limited number of bacterial species, including *Lactobacillus plantarum* [68], and *Selenomonas ruminantium* [69] ferment 2dDR, although these two species should not be a concern for wound dressings as they are not found in skin or in wounds. 

In addition, *Escherichia coli* and *Salmonella enterica* are capable of utilising the 2-deoxy-d-ribosyl moiety of 2′-deoxyribonucleosides as a carbon source [70]. This limited utilisation of 2dDR and 2dDR moieties leads us to believe that a dressing containing 2dDR ought not to encourage any bacterial growth when applied; however, further studies to investigate this are underway in our laboratories.

As above, a wound dressing ideally should not encourage growth of the microbiota either in an infected wound or on surrounding skin. To that end, experiments are underway in our laboratories to measure the utilisation of a broader range of skin dwelling bacteria and wound pathogens, including *Staphylococcus epidermidis*, *Enterococcus faecalis* and the anaerobe *Cutibacterium acnes.* The response of the bacteria to 2dDR both as a sole substrate and as released by the dressing is being investigated in mono- and mixed species. To date, addition of 2dDR to media appears to have either no effect or a negative effect on growth of any species tested (Figure 6B).

### 9.2. Stability and Sterilisation

For sterilisation, our group demonstrated that gamma irradiation was very effective without affecting the stability of 2dDR [33,34] but further work is ongoing to establish protocols for sterilisation and storage of cotton fibre-based dressing containing 2dDR.

## 10. Conclusions

First identified in the structure of DNA in the 1930s, and then as a degradation product of it due to TP activity, 2dDR has more recently been investigated as a stimulator of angiogenesis. Other small sugars have been reported to be pro-or anti-angiogenic, and in particular, of the two isomeric forms of this sugar, the D-form appears to be predominantly pro-angiogenic and the L-form to be anti-angiogenic. While it remains difficult to propose any simple structure–activity relationship between these sugars and their angiogenic activity, the biological response to 2dDR is strong and clearly demonstrable in a series of in vitro and in vivo bioassays, and in animal experiments of cutaneous wound healing.

Incorporation of 2dDR into a variety of experimental and commercial dressings showed good results in all cases. The sugar can be loaded and released over a few days at a concentration which drives angiogenesis in the CAM bioassay and accelerates wound healing in rats.

Its biological profile mimics that of the addition of VEGF with 2dDR achieving 80–100% of the biological actions of VEGF. The sugar is more stable and inexpensive than VEGF, and preliminary results suggest that it can be stored in a variety of potential dressings.

We suggest that the addition of 2dDR to conventional, well-accepted wound dressings may provide a low-cost and simple-to-use pro-angiogenic dressing for use in the treatment of chronic non-healing wounds. This review aims to contribute to the field of angiogenesis by summarising the latest knowledge on a novel pro-angiogenic agent, 2dDR, and introducing it to the scientists working in this field to encourage them to contribute to the further exploration of 2dDR as a pro-angiogenic agent.

## Figures and Tables

**Table 1:** Summary of 2dDR stability studies.

2dDR Was Prepared in	Concentration	Presence of Cells	Remaining 2dDR (%)	Reference
Water	1, 2, 3 mg/mL	No	>95	[33]
Growth Medium	0.135 mg/mL	No	>70	[32]
Growth Medium	0.135 mg/mL	Yes	>60	[32]
